# Membrane Association and Catabolite Repression of the *Sulfolobus solfataricus* α-Amylase

**DOI:** 10.3390/microorganisms3030567

**Published:** 2015-09-18

**Authors:** Edith Soo, Deepak Rudrappa, Paul Blum

**Affiliations:** School of Biological Sciences, University of Nebraska-Lincoln, 1901 Vine Street, Lincoln 68588, NE, USA; E-Mails: edith_soo@yahoo.com (E.S.); deepakgr@unl.edu (D.R.)

**Keywords:** *Sulfolobus*, archaea, α-amylase, secretion, catabolite repression

## Abstract

*Sulfolobus solfataricus* is a thermoacidophilic member of the archaea whose envelope consists of an ether-linked lipid monolayer surrounded by a protein S-layer. Protein translocation across this envelope must accommodate a steep proton gradient that is subject to temperature extremes. To better understand this process *in vivo*, studies were conducted on the *S. solfataricus* glycosyl hydrolyase family 57 α-Amylase (AmyA). Cell lines harboring site specific modifications of the *amyA* promoter and AmyA structural domains were created by gene replacement using markerless exchange and characterized by Western blot, enzyme assay and culture-based analysis. Fusion of *amyA* to the *malA*p promoter overcame *amyAp*-mediated regulatory responses to media composition including glucose and amino acid repression implicating action act at the level of transcription. Deletion of the AmyA Class II *N*-terminal signal peptide blocked protein secretion and intracellular protein accumulation. Deletion analysis of a conserved bipartite C-terminal motif consisting of a hydrophobic region followed by several charged residues indicated the charged residues played an essential role in membrane-association but not protein secretion. Mutants lacking the C-terminal bipartite motif exhibited reduced growth rates on starch as the sole carbon and energy source; therefore, association of AmyA with the membrane improves carbohydrate utilization. Widespread occurrence of this motif in other secreted proteins of *S. solfataricus* and of related *Crenarchaeota* suggests protein association with membranes is a general trait used by these organisms to influence external processes.

## 1. Introduction

The archaeon *Sulfolobus solfataricus* is a thermoacidophilic member of the phylum *Crenarchaeota*. These organisms have unique envelopes including a cytoplasmic membrane comprised of a lipid monolayer surrounded by a glycosylated protein S-layer. Unlike bacteria, they do not have a cell wall made of peptidoglycan. Experimental and bioinformatic studies indicate protein translocation across archaeal cytoplasmic membranes can occur via the Secretory (Sec) pathway and the Twin-Arginine Translocation (TAT) pathway [1,2,3]. Proteins translocated through these pathways require *N*-terminal signal peptides for recognition and targeting to membrane-associated translocation components [4]. Signal peptides are categorized into different classes based on signal peptidase recognition sites [5]. Class 1 constitutes the most common signal peptide found in archaea and is associated with substrates of the Sec and Tat pathways [4]. Class 2 signal peptides contain a conserved motif ([I/L/G/A]-[A/G/S]-C) called a lipobox in which the terminal cysteine undergoes lipidation prior to signal peptide removal to enable protein attachment to the membrane. However, data on the functionality of this class of signal peptides are as yet lacking from studies on archaeal taxa [5]. Class 3 signal peptides have a type IV prepilin-like cleavage site. This class of signal peptides has a unique cleavage site located between the *N*-terminal end and the signal peptide hydrophobic domain [4,5,6]. Class 3 signal peptides have been identified in archaeal flagellins and sugar-binding proteins in *S. solfataricus* [7,8,9].

In eukaryotes and bacteria, Sec-mediated protein translocation can occur using co-translational or post-translational mechanisms [1,10,11]. Co-translational translocation requires the signal recognition particle (SRP) to recognize nascent proteins and target them to the membrane associated Sec translocase [1,10] where the protein is fully translated. Post-translational translocation requires complete synthesis of the protein prior to translocation. SecB acts as a chaperone to prevent stable folding of the nascent protein and targets it to SecA, an energy-utilizing motor domain that is essential for protein secretion [1,10]. The absence of SecA from archaeal genomes implicates a greater role for SRP in the translocation process. In the TAT pathway, proteins are translocated post-translationally in the folded form [12,13]. The pathway is so named because the signal sequence of TAT substrates contains two contiguous arginines [11,12]. The TAT components consist of TatA, which functions as a membrane pore, while TatB and TatC are involved in protein targeting to TatA [1,9]. In *S. solfataricus*, ~1.6% of the proteome (46 proteins) is predicted to consist of putative secretory proteins with *N*-terminal signal peptides [13], suggesting there should be prominent pathways to translocate proteins into or across the cytoplasmic membrane [5]. Although homologs of SRP complex were identified, homologs of SecA and SecB were not present [1,3]. At the same time, while three copies of TatA and two copies of TatC were found in the genome, only five putative TAT substrates were evident [9,11]. Despite these considerations, the main pathway for protein secretion is likely to involve the Sec translocon, yet better model substrates could promote studies to clarify this process.

One such model protein is the *S. solfataricus* homodimeric α-amylase (AmyA) [14]. AmyA is one of only several proteins shown to be fully translocated across the cytoplasmic membrane [14,15,16,17]. AmyA is an endo-acting glycosyl hydrolase, that cleaves starch, dextrin and α-cyclodextrin at 1,4-glycosidic linkages generating linear maltodextrins [14,18]. It belongs to the glycosyl hydrolase Family 57 (GHF 57) based on internal sequence homology [19], including conservation of three amino acids (E506, D609, and E611) that are catalytic residues in other GHF 57 members [20,21,22]. GHF 57 members include α-amylases, 4-α-glucanotranferases, amylopullulanases, and α-galactosidases; with most of these enzymes being found in thermophilic organisms [22]. GHF members from extremophiles have gained significant scrutiny in recent years due to their intrinsic tolerance to high temperature and extreme pH conditions [23]. Despite these useful features, enzymes secreted from hyperthermophiles typically achieve only low abundance in culture supernatants [14,24,25,26] necessitating alternative methods of enzyme production. However, in the case of AmyA, heterologous production in foreign hosts was not successful [27]. In previous studies, it was proposed that AmyA secretion in *S. solfataricus* was limited by transcriptional repression of the natural promoter arising through the action of a catabolite repression system [28]. Data presented here demonstrated this hypothesis was correct and thereby established a strategy for addressing questions about AmyA structure and secretion in its natural host.

## 2. Materials and Methods

### 2.1. Archaeal Strains and Cultivation

Archaeal strains and plasmids used in this study are listed in Table 1. *S. solfataricus* strains were grown in the basal salts medium of Allen [29] as modified by Brock [30] at 80 °C and pH 3.0 in screw-cap flasks with aeration as described previously [19,31]. Tryptone, glucose, and starch from potatoes were added at final concentration of 0.2% (w/v). Growth in liquid culture was monitored spectrophotometrically (540 nm).

### 2.2. Molecular Biology Methods and Strain Constructions

All chemicals were obtained from common chemical suppliers unless indicated otherwise. Molecular biology techniques, including DNA cloning, PCR, and plasmid transformation of *Escherichia coli* DH5α, were performed as described previously [32]. Overlap extension PCR (OLEPCR) [33] and DNA sequencing were as described [31]. Mutant strains of *S. solfataricus* were constructed by markerless exchange [34]. Strains and plasmids (Table 1) and primers (Table S1 supplementary material) are listed.

The *malAp*::*amyA* (*SSO1172*) promoter fusion strain PBL2058 was constructed using plasmid pBN1062. A fragment containing a fusion between *SSO1171* and *malAp* was obtained by PCR of pBN1081 using primers 1171-BamHI-F and MalAp-1172-OLE-R. Plasmid pBN1081 contained a 572 bp fragment extending 107 nt upstream of *SSO1171* through 465 nt of the *SSO1171* open reading frame. This region was fused to a 425 bp fragment encoding *malAp* that spanned regions 425 nt upstream of the *malA* start codon through 7 nt after the *malAp* transcription start site. These fused fragments were joined to the *amyA* open reading frame. Fragments encoding the wild type allele of *amyA* were obtained by PCR using primers MalAp-1172-OLE-F and 1172-BamH1-R. The *SSO1171*::*malAp* fusion to the *amyA* start codon was created by OLEPCR using amplicons encoding the *SSO1171*::*malA*p fusion and *amyA* with primers MalAp-1172-OLE-F and MalA-1172-OLE-R. The resulting *SSO1171*::*malAp*::*amyA* construct was amplified using primers 1171-BamHI-F and 1172-BamHI-R, inserted into the BamHI site of pPB1035 and integrated at *amyA* by markless exchange to create strain PBL2058. PCR and DNA sequencing confirmed the genotype of the *amyA* locus in strain PBL2058. PCR amplification of the *amyA* wild-type allele with primers SSO1171A and ApuPromR2 produced a 550 bp fragment while PCR of the recombinant allele with the same primer pair produced a 1 kb fragment.

**Table 1:** Archaeal strains and plasmids.

	Genotype	Source Or Derivation
**Strains**		
PBL2004	*amyA*::*lacS*	PBL2002 [19]
PBL2025	*amyA +* Δ*(SSO3004-3050)*	PBL2000 [35,36]
PBL2058	*malA*p::*amyA*	PBL2025 by markerless exchange (ME)
PBL2059	*malA*p::*amyA* (*G877Stop)*	PBL2058 by ME
PBL2064	*malA*p::*amyA* Δ*(I2-C31)*	PBL2058 by ME
PBL2065	*malA*p::*amyA* (*K898Stop)*	PBL2058 by ME
**Plasmids**		
pUC19	*bla*	New England BioLabs
pPB1035	*lacS*-KpnI	pUC19 [34]
pBN1081	*1171::malAp::amyA* (fusion at M9)	pUC19 (this work)
pBN1062	*malA*p*::amyA*	pPB1035 (this work)
pBN1063	*amyA (G877Stop)*	pPB1035 (this work)
pBN1064	*amyA* Δ*(I2-C31)*	pPB1035 (this work)
pBN1065	*amyA (K898Stop)*	pPB1035 (this work)

Strain PBL2059 encodes the *amyA* G877Stop allele and was derived by markerless exchange using plasmid pBN1063 (*amyA*G2692T) and strain PBL2058. The *amyA*G2629T fragment was amplified using primers 1172-CTER-SbfI-LF and 1172-CTER-XmaI-RR. The G2629T mutation in *amyA* was created by OLEPCR with primers 1172-CTER-LR and 1172-CTER-RF; both primers encode a T, instead of a G at nt 2629 of *amyA,* thereby removing a diagnostic *Nco*I site. Plasmid PB1063 was constructed by insertion of an *amyA*G2629T amplicon into the *Sbf*I and *Xma*I sites of PB1035. PCR of *amyA* alleles from PBL2025 and PBL2059 using primers 1172-CTER-SbfI-LF and 1172-CTER-XmaI-RR produced a single 1.2 kb fragment. Only DNA amplified from the wild type was cut by *Nco*I and produced two nearly identical fragments (607 bp and 613 bp). DNA sequencing of the PCR amplicons distinguished *amyA* alleles.

Strain PBL2064 encodes an *N*-terminal signal peptide deletion mutation of *amyA* (*amyA* Δ (nt 3-93) and was derived from PBL2058 using plasmid pBN1064. Primers used to amplify the *amyA* 5′ deletion were 1172-NTER-XmaI-LF and 1172-NTER-SphI-RR. The internal deletion of *amyA* was created by OLEPCR using primers 1172-NTER-LR and 1172-NTER-RF and removed a diagnostic *Dde*I site from within *amyA*. Plasmid pBN1064 was created by inserting an *amyA* Δ (nt 3-93) amplicon into *Xma*I and *Sph*I sites of pPB1035. PCR of the wild-type *amyA* allele from PBL2025 with primers 1172-NTER-XmaI-LF and 1172-NTER-SphI-RR produced a fragment of 1.2 kb that was cleaved by *Dde*I into two fragments (647 bp and 555 bp). PCR of the *amyA* Δ (nt 3-93) allele from PBL2064 produced fragment of 1.1 kb that could not be digested using *Dde*I. DNA sequencing verified the identity of the mutant *amyA* allele in strain PBL2064. The *amyA* A2692T recombinant strain, PBL2065, was derived from strain PBL2058 using plasmid pBN1065. The *amyA* A2692T allele was amplified using primers 1172-CHR-SbfI-LF and 1172-CHR-XmaI-RR. The A2692T mutation was created by OLEPCR using primers 1172-CHR-LR and 1172-CHR-RF that encode a T (instead of A), 12 nt upstream of the *amyA* stop codon. Plasmid pBN1065 was constructed by inserting *amyA* A2692T amplicon into the *Sbf*I and *Xma*I sites of plasmid pPB1035. PCR using primers 1172-CHR-SbfI-LF and 1172-CHR-XmaI-RR of both wild type and *amyA* Δ (nt 3-93) alleles produced amplicons of 1.0 kb. DNA sequencing was used to distinguish these alleles of *amyA*. 

### 2.3. Protein Purification

AmyA was purified from clarified culture supernatants as described previously [26]. Culture supernatants were fractionated by passage through columns prepared using High-S cationic resin (Biorad) and equilibrated with 10 mM sodium acetate buffer (pH 3.5). The supernatant was loaded at 1.5 mL/min and columns were washed with sodium acetate buffer at 0.3 mL/min. Bound proteins were eluted at 0.3 ml/min with a 100-mL linear gradient of 0 to 1 M sodium chloride in sodium acetate buffer. Fractions were collected in 3-mL volumes and assayed. Active fractions were concentrated 30-fold by centrifugation using Centricon YM-30 filters (EMD Millipore, Massachusetts, MA, USA).

### 2.4. Α-Amylase Activity Assay

α-Amylase activity was determined using the dextrinization assay [35] with modifications as previously described [19]. Culture supernatants (20 mL) were harvested by centrifugation (7700× *g*) for 15 min to remove cells and then concentrated 20-fold by centrifugation using Centricon YM-30 filters (EMD Millipore, Massachusetts, MA, USA). Cells were harvested by centrifugation (7700× *g*) for 15 min, resuspended in basal salts medium, and lysed by sonication as described [19]. Reaction mixtures containing 0.1 mL of concentrated culture supernatant or whole cell extracts were combined with 100 μg of Zulkowski starch (Fluka) in 10 mM sodium acetate (pH 3.5) and incubated at 80 °C for 120 min. The reaction was terminated by equilibrating the mixture to room temperature. Color was developed by addition of 0.005 mL of an iodine solution (4% (w/v) potassium iodide, 1.25% (w/v) iodine). The sample absorbance was determined at a wavelength of 600 nm and was corrected for an identical sample, but without incubation. All assays were performed in duplicate and the average of the results are reported. One unit of activity was equivalent to the amount of protein which hydrolyzed 1µg of starch in 1 min.

### 2.5. Starch Plates Diffusion Assay

Starch plate diffusion assays used a solid medium containing basal salts and 0.6% (w/v) Gelrite (Kelco) and magnesium chloride (at a final concentration of 8 mM) as described previously [19]. Starch, tryptone, or glucose were added at a final concentration of 0.2% (w/v). Strains were grown in tryptone medium or minimal glucose medium to mid-exponential phase, and a sample equivalent to 1 × 10^8^ cells was applied to the surface. Plates were incubated at 80N ^o^C for 3 days and Gram iodine solution (Thermo Fisher Scientific, Waltham, MA, USA) was added to the plates to visualize zones of clearing due to starch hydrolysis.

### 2.6. Sub-Cellular Fractionation

Cells were harvested by centrifugation (7700× *g*) for 15 min, and resuspended in 20 mM cacodylate buffer (pH 6.0) containing 10 mM NaCl, 0.5 mM MgSO_4_, and 1 mM phenylmethylsulfonyl (PMSF) prepared in isopropanol, then lysed by sonication. The S-layer and cell membrane were separated from the cytoplasmic fraction as described previously [27,28,36]. The mixture was centrifuged (at 100,000× *g*) for 30 min in an SW-60 rotor (Beckman Coulter, Inc., Indianapolis IN, USA) to separate cytoplasmic and cell envelope fractions. The pellet containing the envelope fraction was then processed to separate membrane and S-layer components using a combination of 20 mM cacodylate buffer containing 0.5% sodium sarcosyl and 1 mM PMSF (Buffer A) and 20 mM cacodylate buffer containing 0.2% sodium dodecyl sulfate (Buffer B). Samples were resuspended in Buffer A, incubated at 37 °C for 10 min, and centrifuged (7500× g) for 15 min. The supernatant containing the solubilized membrane fraction was collected and the same procedure was repeated again using Buffer A and then twice again using Buffer B. The resulting supernatants were collected and combined. The remaining pellet containing the S-layer, was resuspended successively in 20 mM cacodylate buffer (Buffer C) and then distilled water, incubated at 37 °C for 10 min, and centrifuged (7500× g) for 15 min, then resuspended in Buffer C.

### 2.7. Protein Electrophoresis and Western Blot Analysis

Prior to electrophoresis, samples were adjusted to 2% (w/v) SDS and 3 mM β-mercaptoethanol and boiled for 10 min. Proteins were resolved by SDS-PAGE with 10% (w/v) resolving gels and 5% (w/v) stacking gels, and PageRuler prestained molecular mass standards (Fermentas). Chemiluminescent western blot analysis was performed using the ECL system (Amersham Biosciences, Pittsburgh, PA, USA) as previously described [19,32], with modification. Western blots were probed with 1:5000 dilution of the anti-amylase antibody [19], and then 1:5000 dilution of HRP goat anti-rabbit IgG secondary antibody (Zymed, Rockford, IL, USA). Purified amylase (40 ng) was used as a standard for Western blot analysis. Anti-amylase antibody was pre-adsorped using cell extracts from the *amyA* disruption strain, PBL2004 [19], to remove non-specific antibodies, as previously described [37] with modification. Pre-wetted nitrocellulose membrane (80 μg protein/cm^2^) was incubated with sonicated cell pellets for 3 h at room temperature, and blocked overnight in 2.5% (w/v) milk (Nestle, Glendale, CA, USA) in PBS buffer (10 mM NaH_2_PO_4_ and 150 mM NaCl). Nitrocellulose membranes were washed three times with PBS buffer and incubated with 1:5000 dilution of anti-amylase antibody in 0.5% (w/v) milk in PBS buffer for 2 h at room temperature.

### 2.8. Bioinformatic Analysis

Sequences of putative secreted proteins [5] were obtained from *S. solfataricus* P2 complete genome at NCBI (GenBank Accession number: AE006641). Sequences of putative secreted proteins in other closely related species were derived by BLASTp at NCBI using sequences from *S. solfataricus* strain P2.

## 3. Results

### 3.1. Promoter Substitution and Catabolite Repression

**Figure 1:**
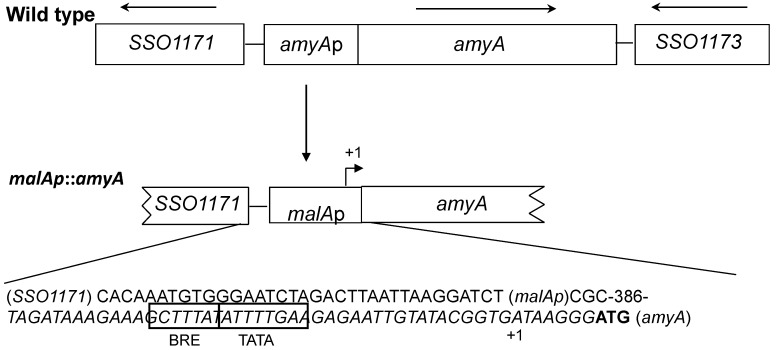
The *amyA* locus, promoter fusion. Top, PBL2025 (wild type), *amyA* (*SSO1172*) and flanking ORFs. Bottom, PBL2058 (*malAp*::*amyA*) promoter substitution using *malAp*. DNA sequence of fusion junctions for *SSO1171*::*malAp* and *malAp*::*amyA* are shown. The *malAp* promoter elements (BRE, TATA) are boxed, *malAp* transcription start is indicated (+1), *amyA* start codon is shown in bold.

Previous studies on the *S. solfataricus* α-amylase, AmyA, had demonstrated that levels of this secreted protein were responsive to the carbon composition of the medium, a response that constituted part of a catabolite repression-like system [14,28]. If changes in AmyA abundance arose from transcriptional regulation, they might obscure *in vivo* studies designed to study the role of AmyA motifs on protein function and secretion. To address possible transcriptional regulation, the *amyA* promoter was substituted using an alternative promoter. An *amyA* promoter fusion strain was constructed and characterized using the constitutive *malA*p promoter [38]. The *malAp* sequence including a 7 nt untranslated leader RNA were fused to the first internal methionine codon in the *amyA* reading frame replacing *amyA* sequences thought to encode the natural promoter (Figure 1).

The promoter fusion cassette was cloned into pUC19 along with 5′ flanking sequences from SSO1171 and 3′ sequences from *amyA* to provide regions for homologous recombination at the *amyA* locus. The promoter fusion was then integrated into the chromosome by homologous recombination via markerless exchange [34], thereby replacing the endogenous *amyA* promoter to create strain PBL2058 (Figure 1). Production of AmyA was then examined during growth on various media to assess the role of catabolite repression.

**Figure 2:**
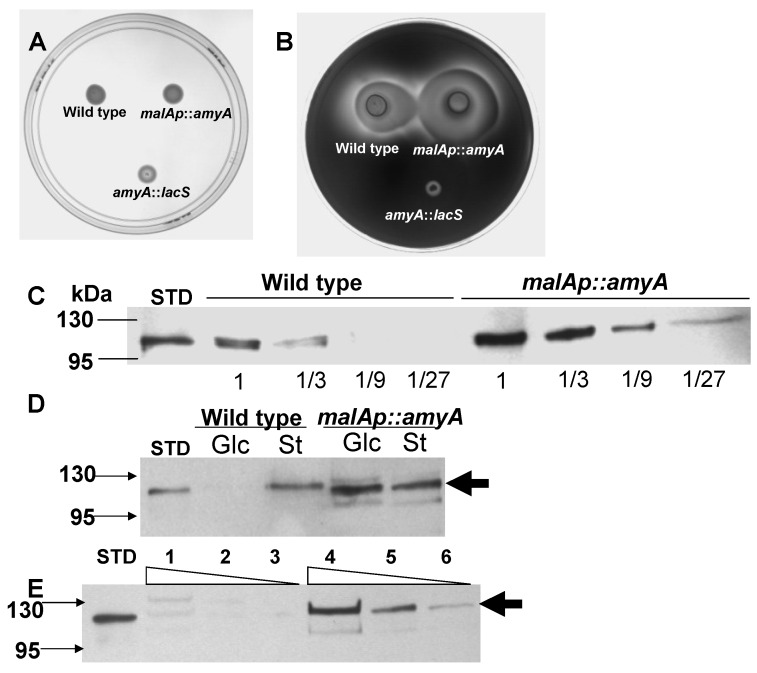
Effect of promoter substitution on amino acid and glucose repression. Starch diffusion plate assay and western blot analysis of AmyA levels culture supernatant grown in a medium with Tryptone. **Panel A**, no treatment. **Panel B**, Iodine treatment. Numbered spots were: 1 (wild type, PBL2025); 2 (*malA*p-*amyA*, PBL2058); 3 (*amyA*::*lacS*, PBL2004). All spots contained 10^7^ cells and plates were incubated at 80 °C for 6 days. **Panel C**, three-fold serial dilutions of wild type (PBL2025) culture supernatant and three-fold serial dilutions of the *malA*p::*amyA* promoter fusion strain (PBL2058) culture supernatants. In Panel C, purified AmyA standard (STD, 40 ng) is indicated. Lane 1 in both panels contains protein dervived from a culture volume proportional to 10^9^ cells. **Panel D**, western blot analysis of culture supernatants from PBL2025 (wild type) and PBL2058 (*malAp*::*amyA*) strains grown in a medium containing either glucose (Glc) or starch (St) as sole carbon and energy sources respectively. Samples of culture supernatants were equivalent to a volume proportional to 5 × 10^9^ cells. **Panel E**, western blot analysis of three-fold serial dilutions of supernatants from PBL2025 (wild type) grown in glucose (lanes 1–3) or starch (lanes 4–6) as sole carbon and energy sources. Purified AmyA (STD, 40 ng) was used as a standard. Lanes 1 and 4 contain protein dervived from a culture volume proportional to 6 × 10^8^ cells.

**Figure 3:**
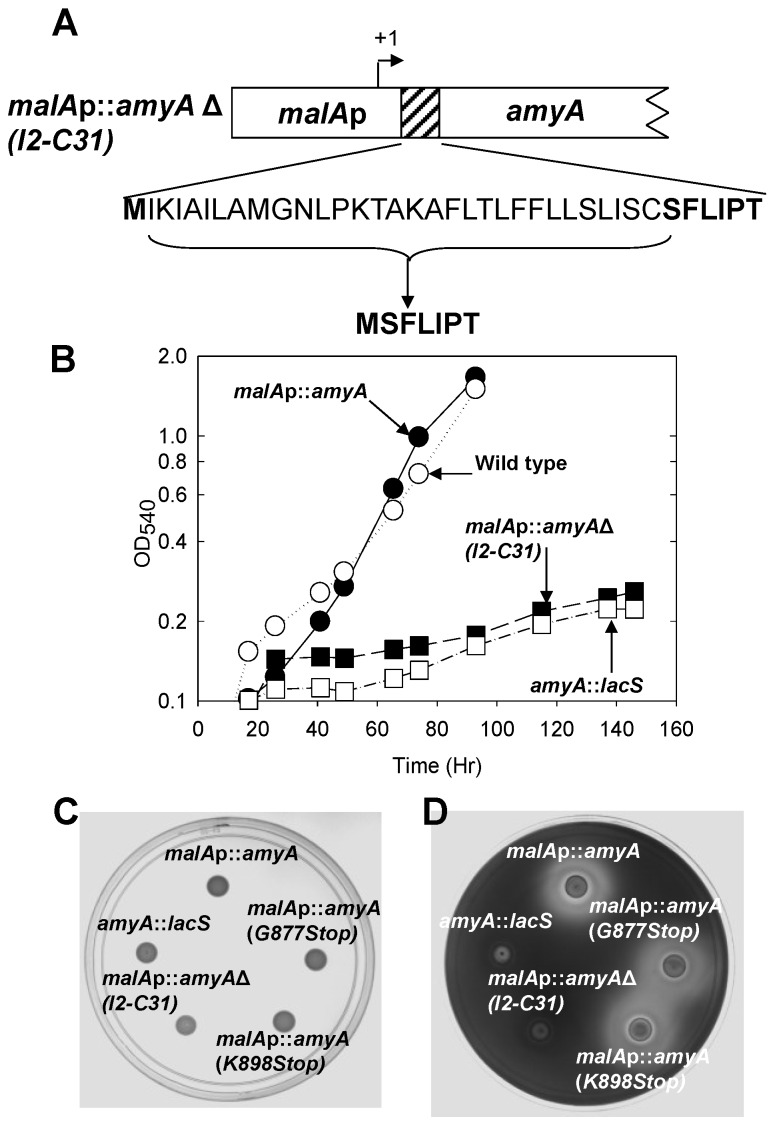
**Panel A**, The *amyA* locus, leader sequence deletion. PBL2064 (AmyA leader peptide deletion), deleted amino acids are bracketed, remaining amino acids are shown in bold. **Panel B**, Efficiency of starch utilization during growth in liquid culture. Strains were; leader peptide mutant (PBL2064, closed squares), wild type (PBL2025; open circles), *malAp*::*amyA* promoter fusion (PBL2058, closed circles) and *amyA*::*lacS* disruption (PBL2004, open squares). **Panels C** and **D**, Amy A production during growth on a solid medium. **(a)** no treatment. **(b)** Iodine treatment. Numbered spots were: 1 (*malA*p-*amyA*, PBL2058), 2 (Gly877Stop mutant (PBL2059), 3 (Lys898Stop mutant, PBL2065), 4 (*N*-terminal deletion mutant, PBL2064), 5 (*amyA*::*lacS* disruption mutant, PBL2004). All spots contained 10^7^ cells and plates were incubated at 80 °C for 6 days.

AmyA synthesis is also responsive to a carbon source hierarchy and can be repressed by addition of specific amino acids or complex carbon sources such as tryptone [14,39]. Therefore the impact of the *malAp* promoter fusion on amino acid repression of *amyA* expression was determined. Diffusion assays were performed using a solid medium containing both starch and tryptone as carbon and energy sources. After 6 days of incubation at 80 °C, the plates were developed by iodine treatment. A larger zone of clearing due to secreted α-amylase was observed for PBL2058, relative to the parental strain PBL2025 (Figure 2A,B). In contrast, there was no zone of clearing for the *amyA*-*lacS* disruption strain PBL2004 [19]. Western blot analysis using culture supernatants was performed to determine the level of total secreted AmyA produced by strains PBL2058 and PBL2025. The strains were grown in a liquid medium with added tryptone and starch. Culture supernatants from cells in exponential phase were concentrated and 3-fold serial dilutions were examined with the least dilute sample equivalent to a culture volume harboring 1 × 10^9^ cells. Under these growth conditions, levels of secreted AmyA produced by the promoter fusion strain PBL2058 were at least six-fold higher than those of the wild type strain (Figure 2C). These results indicate amino acid repression uses a transcriptional mechanism to control *amyA* expression and that the *malAp*::*amyA* promoter fusion is insensitive to this form of catabolite repression. As secretion of AmyA into the culture supernatant had also been shown to respond to glucose repression [14], Western blot analysis was performed using culture supernatants from cells grown using glucose or starch as sole carbon and energy sources. Culture supernatants harvested during exponential phase were concentrated and examined. Levels of AmyA produced by the wild type strain varied significantly during growth on glucose; while levels in the promoter fusion strain were unaffected by medium composition (Figure 2D). Growth rates and cell yields of strain PBL2058 in a defined liquid medium using starch as the sole carbon and energy source were identical to those of the parental strain in (PBL2025) indicating the promoter fusion construct was active (Figure 3B).

These data indicate a second form of catabolite regulation, called glucose repression, depends on the *amyA* promoter, and that replacement of this sequence by *malAp* provides a catabolite insensitive cell line for AmyA synthesis. *In vivo* promoter substitution experiments also provided a measure of the degree of difference in AmyA levels during growth on glucose versus starch minimal media. Levels of AmyA produced by the wild type strain during growth on starch versus glucose as sole carbon and energy sources differed by nine-fold (Figure 2E). The magnitude of this effect observed here using Western analysis of strain PBL2025 is similar to the 10-fold effect reported previously using enzyme assays and *Sso* strain 98/2 [14]. Subsequent manipulations of *amyA* were constructed in the promoter fusion strain PBL2058 to facilitate *in vivo* characterization in the absence of catabolite repression control. 

### 3.2. Role of the Class II Amya Leader Sequence

A whole genome survey of leader peptide distribution in *Sso* reported that SSO1172 (later identified as *amyA* [19]) encoded a Class II family leader peptide [5]. While *in vitro* data have demonstrated cleavage of Class III leader peptides [40], the *in vivo* role for leader peptides of any class has not been established in this organism. Therefore, the consequence of leader peptide deletion on AmyA production was examined. The *malAp*::*amyA* fusion construct was modified *in vitro* by overlap PCR to remove sequences encoding the entire leader peptide along with the putative signal peptidase cleavage site. The start codon was retained in the modified construct to ensure faithful translation initiation (Figure 3A). Following gene replacement by maker less exchange of the endogenous *amyA* gene with the *N*-terminal deletion construct, the growth of the resulting strain, PBL2064, was evaluated relative to controls in a liquid minimal medium containing starch as the sole carbon and energy source. Strain PBL2064 was unable to grow under these conditions (Figure 3B). Levels of AmyA activity in whole cell-extracts and in culture supernatants of cultures grown in a defined minimal medium containing glucose as the carbon and energy source were undetectable relative to controls (Table 2).

To uncouple the relationship between growth and secretion, starch plate diffusion assays were conducted using a complex medium. No zone of starch degradation was detected (Figure 3C,D), suggesting AmyA production was defective in strain PBL2064.

**Table 2:** α-Amylase activity in selected strains.

Strain	α-Amylase Activity * (U/mL)	
	Supernatant	Cell Extracts
AmyA^+^ (PBL2025)	0.23 ± 0.01	0.13 ± 0.02
*malAp::amyA* (PBL2058)	0.16 ± 0.01	0.18 ± 0.01
*malAp::amyA* G^877^ to STOP (PBL2059)	0.17 ± 0.01	0.03 ± 0.01
*malAp::amyA* K^898^ to STOP (PBL2065)	0.15 ± 0.01	0.02 ± 0.01
*malAp::amyA* Δ I^2^ to C^31^ (PBL2064)	<0.01	0.01
AmyA^−^ (PBL2004)	0.02 ± 0.03	0.02 ± 0.06

* One unit of activity equals 1µg of starch hydrolyzed per min per mL of sample. Samples were analyzed at cell densities of 10^9^/mL.

### 3.3. Role of the Amya C-Terminal Motif

**Figure 4:**
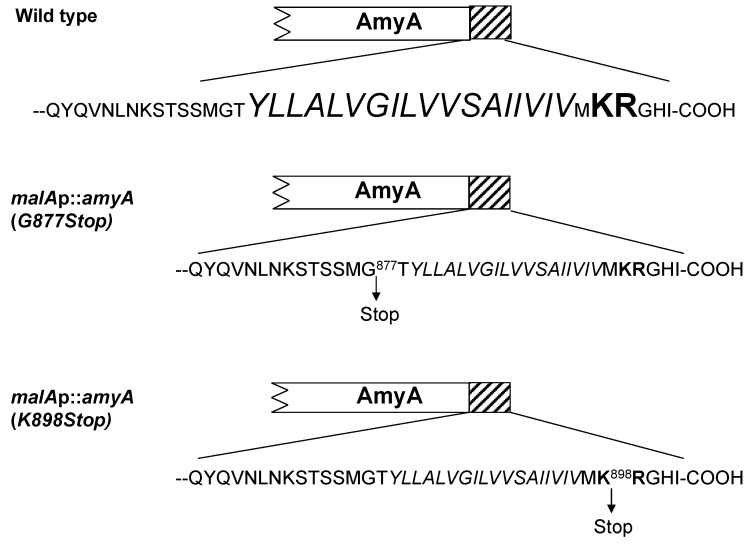
Modification of the AmyA C-terminal bipartite motif. Top, PBL2025 (wild type). The C-terminal motif is shown, residues within the hydrophobic domain are italicized, and charged residues are bold. Center, PBL2059 (deletion of entire C-terminal motif). Gly877 was mutagenized to create a nonsense mutation. Bottom, PBL2065 (deletion of 5 terminal residues including two charged positions). Lys898 was mutagenized to create a nonsense mutation.

The C-terminal end of AmyA contains a stretch of 18 hydrophobic residues followed by an additional five residues of which two are charged including Lys^898^ and Arg^899^ (Figure 4). This bipartite C-terminal motif, a hydrophobic region followed by several charged residues, was conserved in other *S. solfataricus* proteins annotated as secreted (Table S2). Examination of additional putative secreted proteins from *Metallosphaera sedula* and other *Sulfolobus* species revealed this same bipartite C-terminal motif was conserved (Table S2). To examine the role of this motif, two additional *S. solfataricus* strains were constructed that had distinct nucleotide substitutions in *amyA* resulting in premature translation termination codons that would truncate protein length and remove all or part of the bipartite motif. Strain PBL2059 was constructed by introducing a point mutation at nt 2629 of *amyA* converting Gly^877^ into a stop mutation and resulting in loss of both the hydrophobic region and the C-terminal charged residues (Figure 4). PBL2065 was constructed by introducing a point mutation at nt 2692 converting Lys^898^ into a stop mutation and resulting in loss of only the C-terminal charged residues (Figure 4).

**Figure 5:**
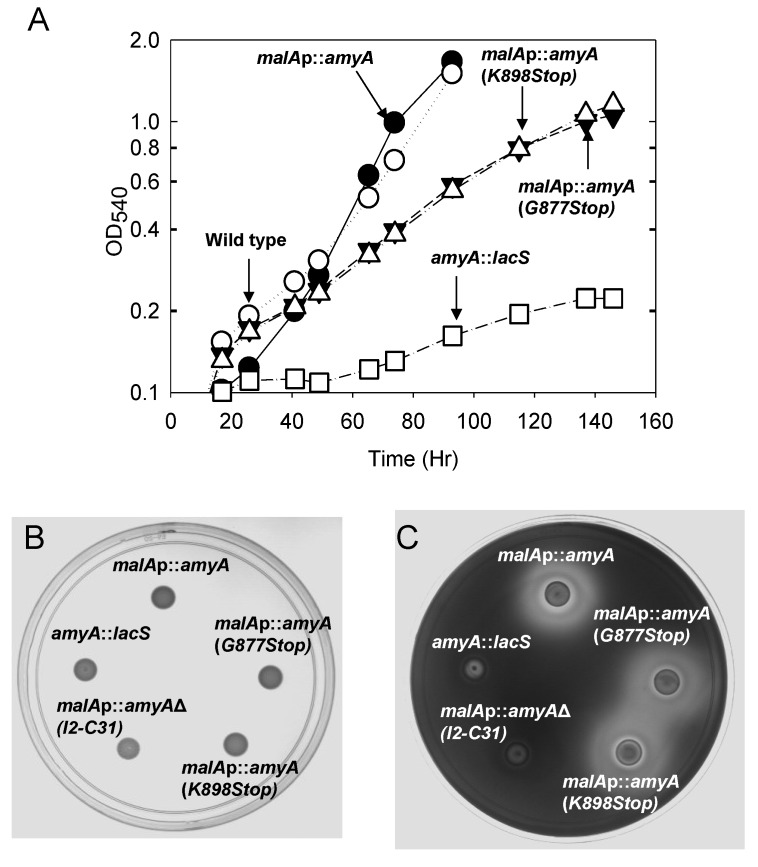
**Panel A**, Efficiency of starch utilization during growth in liquid culture. Strains were grown at 80 °C in batch culture with aeration using a minimal starch medium. Strains were; Gly877Stop mutant (PBL2059, closed triangles), Lys898Stop mutant (PBL2065, open triangles), wild type (PBL2025; open circles), *malAp*::*amyA* promoter fusion (PBL2058, closed circles) and *amyA*::*lacS* disruption (PBL2004, open squares). Amy A production during growth on a solid medium. **Panel B**, no treatment. **Panel C**, Iodine treatment. Numbered spots were: 1 (*malA*p-*amyA*, PBL2058), 2 (Gly877Stop mutant PBL2059), 3 (Lys898Stop mutant, PBL2065), 4 (*N*-terminal deletion mutant, PBL2064), 5 (*amyA*::*lacS* disruption mutant, PBL2004). All spots contained 10^7^ cells and plates were incubated at 80°C for 6 days.

**Figure 6:**
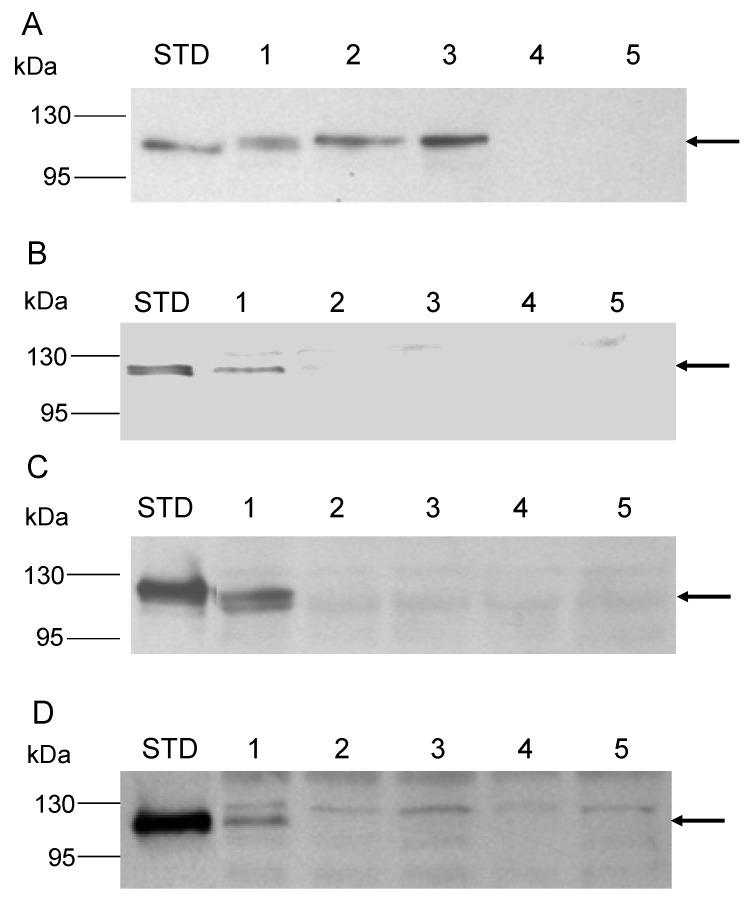
Western blot analysis of supernatant and sub-cellular fractions. **Panel A**, culture supernatants. **Panel B**, cytoplasmic fractions. **Panel C**, membrane fractions. **Panel D**, S-layer fractions. Lanes were; STD (purified AmyA), 1 (*malA*p-*amyA*, PBL2058), 2 (Gly877Stop mutant, PBL2059), 3 (Lys898Stop mutant, PBL2065), 4 (*N*-terminal deletion mutant, PBL2064), 5 (*amyA*::*lacS* disruption mutant, PBL2004). Supernatant samples were derived from culture volumes proportional to 1 × 10^9^ cells.

**Figure 7:**
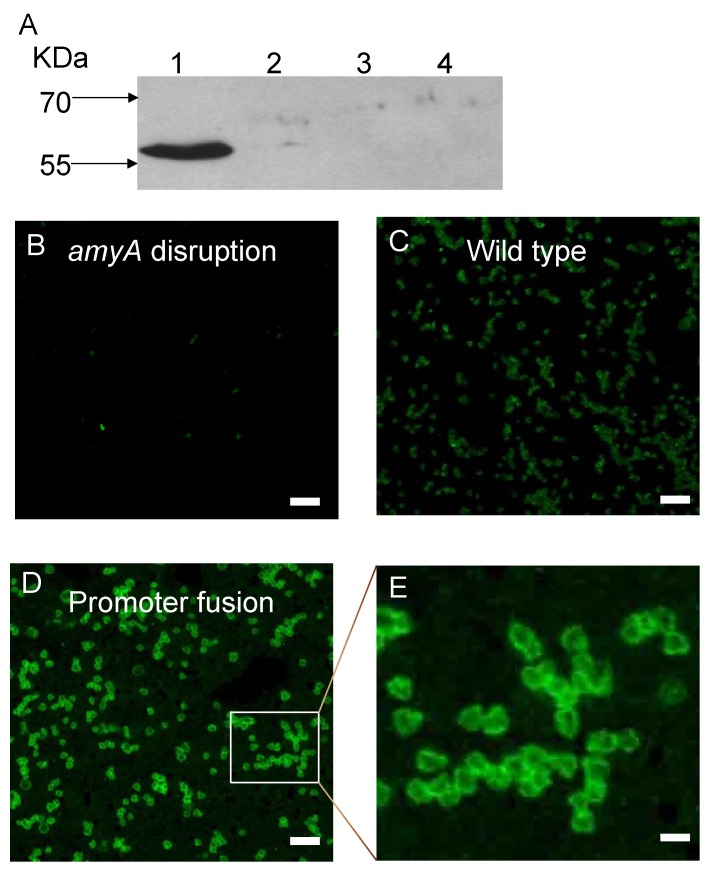
**Panel A**, Western blot analysis of β-glycosidase (LacS) from *S. solfataricus* wild-type strain, 98/2. Samples were: cytoplasmic fractions (Lane 1), membrane fractions (Lane 2), S-layer fractions (Lane 3); and supernatant (Lane 4). Samples were loaded in an amount equivalent to 1.0 OD_540_ of cells. The western blot was probed with a 1:5000 dilution of the anti-LacS antibody, followed by a 1:5000 dilution of HRP goat anti-rabbit IgG secondary antibody. Immunofluorescence analysis of cell-associated α-Amylase. Fixed cells were treated with anti-α-Amylase antibodies conjugated to Alexafluor dye. **Panel B**. Cells lacking the α-amylase gene. **Panel C**. Wild type cells. **Panel D**. Cells encoding the α-amylase promoter fusion. **Panel E**. Inset of panel C. Bars in Panels A–C 5 microns, bar in panel D 0.5 microns.

Growth of the two strains was compared to controls using a defined minimal starch medium. Strains PBL2025 (*amyA*^+^) and PBL2058 (*malAp*::*amyA*) were included as positive controls, while strain PBL2004 (*amyA*::*lacS*) was included as a negative control. Both strains PBL2059 and PBL2065, containing the C-terminal truncated versions of AmyA exhibited a 50 percent decreased rate of growth relative to controls (Figure 5A). In contrast, starch diffusion plate assays using a starch medium supplemented in this case with glucose to support growth, showed that both strains producing C-terminal truncated AmyA proteins exhibited diffusion zones-of-clearing that were comparable to controls (Figure 5B,C). In addition, purified AmyA from strains PBL2025 (*amyA*^+^) and PBL2059 (Gly^877^Stop) had specific activities of 21.8 U/mg and 22.4 U/mg, respectively, thereby excluding an effect of the loss of the C-terminal motif on enzyme function. α-Amylase assays of cultures grown in a defined minimal glucose medium demonstrated there was active α-amylase activity in the culture supernatants of both strains PBL2059 and PBL2065 at levels comparable to the positive controls (Table 2). In contrast, levels of α-amylase activity were very low in whole cell extracts of these strains again comparable to those observed in samples from the negative control (Table 2). As it remained possible that leader-deficient AmyA was present but could not dissociate from intact cells, Western blot analysis of sub-cellular fractions was performed to verify this. AmyA was not detected in the cytoplasm, membrane or S-layer fractions relative to control samples (Figure 6A–D , lane 4). In contrast, AmyA was evident in all fractions derived from the wild type strain (Figure 6A–D, lane 1). Association of AmyA with the membrane and S-layer is consistent with earlier studies reporting presence of cell-associated AmyA activity [14].Western blot analysis of AmyA abundance in these samples indicated levels were undetectable in fractions derived from the cytoplasm, the cell membrane or the S-layer of the two AmyA C terminal truncated strains (Figure 6A–D). However, AmyA was evident in the culture supernatants of both strains at levels similar to those observed in samples from the positive controls (Figure 6A). As similar results were obtained using both types of AmyA truncations, the conserved C-terminal charged residues are apparently required to ensure continued membrane association but play no role in protein secretion. To ensure the efficiency of the cellular fractionation procedure, the cytoplasmic β-glycosidase, LacS, was used as a diagnostic marker for cytoplasmic contamination by Western blot analysis (Figure 7A). LacS was evident only in the cytoplasmic fraction (lane 1) and was not detectable at equivalent mass loadings in either the membrane or S-layer fractions (lanes 2, 3). To verify the cell-associated presence of α-Amylase immunofluorescence analysis of whole cells was conducted using fluorophore conjugated anti-α-Amylase antibodies (Figure 7B–E). Immunofluorescence signal was largely absent from cells incapable of producing α-Amylase due to disruption of *amyA* (panel B) but readily evident in the wild type (panel C) and cells overproducing α-Amylase as a result of promoter fusion to *malAp* (panel D; and inset panel E). These data verify the localization of α-Amylase as a cell-associated surface exposed protein.

## 4. Discussion

In this study, a novel bipartite C-terminal motif present in the secreted α-amylase (AmyA) of *S. solfataricus* was identified and its role in membrane localization established. The bipartite motif consists of a hydrophobic region followed by several charged amino acids that are usually contiguous. Removal of the charged amino acids *in vivo* by expression of a terminally truncated enzyme was sufficient to block membrane association of AmyA, but not its secretion indicating an essential requirement for these residues. Interestingly, both components of the bipartite motif are widely distributed in secreted proteins of *Sulfolobales* taxa indicating the importance of this region [41]. In unrelated organisms, a hydrophobic region in the C-terminus of translocated proteins can act as a transmembrane anchor component [42,43,44]. In addition, C-terminal charged residues are a retention signal in cell-wall anchored proteins of *Firmicutes* [45]. The charged residues present in the C-terminal motif of AmyA may be recognized by a membrane-bound translocase component that promotes membrane retention of the enzyme. These C-terminal residues may thus function in a related manner to the twin arginine motif recognized by TatC in TAT pathway protein translocation [46,47]. Alternatively, they may act as a proteolytic cleavage site.

In wild type *S. solfataricus*, AmyA is secreted into the supernatant while a minor fraction remains membrane associated and accumulates in the cytoplasm. Ablation of the bipartite motif overcomes membrane association while secretion remains unaffected. Importantly, the loss of membrane association strongly affected the efficiency of starch utilization in a liquid medium thereby compromising growth rate. This suggests that starch hydrolysis occurring in close proximity to the cell facilitates subsequent uptake of starch hydrolytic products and in this regard resembles certain aspects of the sortase system employed by *Firmicutes* to maximize substrate utilization. In *Bacilli*, protein hydrolases can be covalently attached to the external face of the cell wall via action of the enzyme sortase [48]. These proteins contain an *N*-terminal signal peptide that targets the cytoplasmic membrane and a C-terminal cell wall sorting signal consisting of a sortase-recognition motif, LPXTG, hydrophobic domain and a positively charged C-terminus [49,50]. A genetic study in *Staphylococcus aureus* demonstrated that deletion of the hydrophobic domain and positively charged tail abolished cell wall anchoring and the proteins are secreted into the culture supernatant [50]. This suggests that the hydrophobic residues and positively charged tail function to retain the protein within the cell [45,50]. This retention in turn allowed the membrane-associated sortase to recognize the LPXTG motif, and cleave between the threonine and glycine residues [51]. The COOH-terminal threonine then forms an amide bond with the amino group of Lipid II, which is a precursor of the pentaglycine cell wall crossbridge through a transpeptidation reaction. Subsequently, this lipid II-linked protein is incorporated into the growing peptidoglycan during cell wall synthesis, forming a mature cell wall anchored surface protein [52,53]. In archaea, however, both the sortase and the LPXTG substrate motif are not evident, though using C-terminal tripartite sorting signals, potential archaeosortases have been identified [54]. At the same time, archaeal prokaryotes do not have peptidoglycan cell walls. Only the hydrophobic domain and C-terminal charged residues are present and, as proposed here, appear to play related roles in membrane association on the external face. 

It had been proposed that AmyA contained a lipobox-type signal peptide, based on systematic whole-genome analysis [5]. As shown here, deletion of the putative signal peptide demonstrated its necessity for AmyA secretion. Unlike the wild type strain, AmyA did not accumulate in the cytoplasm of the *N*-terminal deletion mutant. This result is in contrast to examples where deletion or mutation of an α-amylase did not perturb cytoplasmic accumulation [55,56,57]. It is possible that *N*-terminal truncation of AmyA reduces its cytoplasmic half-life by analogy to similar manipulations removing leader sequences [58] and Tat signals in bacterial taxa [59].

Previous studies demonstrated that repression of AmyA abundance in the extracellular environment of *S. solfataricus* was controlled by catabolite repression but the mechanism for this effect was not determined [14]. Repression was most acute in a complex medium due to the presence of specific amino acids, notably, aspartic acid, glutamic acid, proline, leucine, and lysine. Repression was also observed during growth on glucose as the sole carbon and energy source, possibly due to the effect of catabolite repression [14]. In this study, both forms of catabolite repression were found to depend on the *amyA* promoter. While amino acid repression had been shown to use a transcriptional regulatory mechanism for other *S. solfataricus* genes [38], the mechanism employed for glucose repression had not been described. Availability of an *S. solfataricus* strain in which AmyA production is catabolite-insensitive enables *in vivo* studies to be conducted independently of the carbon composition of the medium, and facilitates efforts to explore further the role of the bipartite motif as a membrane anchor. 

## Supplementary Files

Supplementary File 1
