# Supplementary material for: Membrane Association and Catabolite Repression of the Sulfolobus solfataricus α-Amylase
Source: Microorganisms. 2015 Sep 18;3(3):567–87. doi: 10.3390/microorganisms3030567 (PMC5023256; doi:10.3390/microorganisms3030567)
Supplement: Supplementary File 1 [file microorganisms-03-00567-s001.docx]

**Supplementary Information**

# Table S1. Conserved C-terminal end motif in putative secreted proteins of *Sulfolobus* and related species.

| **ORF** | **Gene Annotation** | **C-Terminal Motif** ***** |
| --- | --- | --- |
| SSO0011 | S-layer | *I*G*II*T*IIVIIVIIIVIAAVLL*R*V*RNKRR |
| SSO1141 | Protease related protein | *I*S*LAI*G*IIAIVLAILALV*RRRR |
| SSO1886 | Thermopsin precursor | *IVAVIVIAVVAIAILVL*RRRR |
| SSO2045 | Protease | *I*G*IIA*G*IILIIVIVVILVI*SKRK |
| SSO2181 | Peptidase related protein | *IL*T*I*G*IVL*D*II*T*IIALILI*KRRKK*FI* |
| SSO2801 | Cytochrome b558/566, subunit A | *IV*G*VVIAIIALIIL*Y*VVF*RR |
| ***Sulfolobus acidocaldarius*** | | |
| SAC1147 | Peptidase S8 | *LIIAII*S*LILVI*Y*LLL*RRR |
| SAC1183 | S-layer | *LI*Y*VIIAIIIVIIIIVVVL*SR*F*RRKK |
| SAC1534 | Thermopsin | *LVI*P*IV*T*AVVIVVAI*TRRR |
| SAC1760 | ABC-type oligopeptide transporter | *IVAVIVVVIIIIAAVVIL*RRR |
| SAC1858 | cytochrome b558/566 subunit A | *II*G*VVVALVALVIL*Y*VVF*RR |
| SAC2277 | Predicted protease | *LIAAIIALAL*G*IVAIFI*GRRK |
| ***Sulfolobus tokodaii*** | | |
| STO0706 | Extracellular solute-binding protein | *LIAVIVIVIIIIIAVAVVLL*RRR |
| STO1102 | Alpha-amylase | *IL*ST*IL*GS*IVALLIII*E*II*RRRY*I* |
| STO1913 | S-layer domain | *LVIIVVIIIIILIVVVVAL*RSRRKH |
| STO2364 | Hypothetical pseudomonapepsin | *II*G*LVIAIVAVILFLLLLF*RRGGKR |
| STO2534 | ABC-type dipeptide transporter | *LIV*G*VAVAIIIVVIIVAVLLL*RRRR |
| STO2626 | Thermopsin | *VVAILIIVAIVAIVVIV*RRK |
| ***Metallosphaera sedula*** | | |
| MSE0592 | S-layer like domain protein | *AF*Y*IAIVVVIIIIVVVLILL*RRKK |
| MSE1206 | Copper domain protein | *I*SS*L*YS*LL*T*VLIVLVVI*S*LIL*N*VVLIA*RRR |
| MSE1231 | Peptidase A5, thermopsin | G*IVAVIAIVAV*G*VVIVLI*KRR |
| MSE1460 | Thermopsin | *LVIAVIVVIVIWVLI*NR*F*RKPD*L*N*I* |
| MSE1369 | Peptidase S8 and S53, subtilisin, kexin,sedolisin | *V*G*IIALAIVVVILLILVL*R*V*RK |
| MSE0504 | Hypothetical protein (cbsA) | *V*T*VV*G*VIVAIIALLAL*Y*VVF*RR |

* Italicized characters represent hydrophobic residues, and the bolded characters represent charged residues.

**Table S2.** Primers.

| **Primers** | **Sequence (5′–3′)** | **Primer Location** | **Restriction Site** |
| --- | --- | --- | --- |
| 1171-BamHI-F | AGTCAGGATCCCGCGCCTCCAGAATCACTAG | 5′ end starts 443 nt downstream of *SSO1171* | *Bam*HI |
| MalAp-1172-OLE-R | GCTAAAATTGCAATTTTTATCATCCCTTATCACCGTATACAATTCT | 5′ end starts 23 nt upstream of *malA* start codon, followed by *amyA* start codon and 20 nt thereafter | n/a |
| MalAp-1172-OLE-F | AGAATTGTATACGGTGATAAGGGATGATAAAAATTGCAATTTTAGC | complementary to MalA-1171-OLE- R | n/a |
| 1172-BamHI-R | AGTCAGGATCCATAGTATATACCTTGTGGTA | 3′ end starts 919 nt from the *amyA* start codon | *Bam*HI |
| SSO1171A | GATAGAAAAAGGGAAAAGATGGG | 3′ end starts on start codon of *SSO1171* | n/a |
| ApuPromR2 | GGTTGCGCTGAAATTCTGCCCATTCC | 5′ end starts 266 nt downstream of *amyA* start codon | n/a |
| 1172-CTER-SbfI-LF | CCTGCAGGGGAATAATGGATACCTTGGAAAGACTG | 5′ end starts 2018 nt downstream of *amyA* start codon | *Sbf*I |
| 1172-CTER-LR | CAACAGGTAAGTTCACATGGAACTTG | 5′ end starts 89 nt upstream of *amyA* stop codon | n/a |
| 1172-CTER-RF | CAAGTTCCATGTGAACTTACCTGTTG | complementary to 1172-CTER-LR | n/a |
| 1172-CTER-XmaI-RR | CCCGGGGGTTGGATTCATATTTCCCTATTTGATC | 3′ end starts 530 nt downstream of *amyA* stop codon | *Xma*I |
| 1172-NTER-XmaI-LF | GCTAGCCCCGGGCTTTTTCCCTTTTCTACCCAAGGCGAACCCAC | 5′ end starts 427 nt upstream of the *amyA* start codon | *Xma*I |
| 1172-NTER-LR | CTGTGATGTTGGAATGAGGAATGACATCCCTTATCACCGTATACAATTC | 5′ end starts 22 nt upstream of *amyA* start codon, including the start codon; followed by 24 nt from position 91–114 downstream of the *amyA* start codon | n/a |
| 1172-NTER-RF | GAATTGTATACGGTGATAAGGGATGTCATTCCTCATTCCAACATCACAG | complementary to 1172-NTER-LF | n/a |
| 1172-NTER-SphI-RR | GCTAGCGCATGCCCCATTGGCCTGGCTTAAAAGGA CC | 3′ end starts 493 downstream of the *amyA* start codon | *Sph*I |
| 1172-CHR-SbfI-LF | GCTAGCCCTGCAGGGGAATTACCTATATGTTGCAG | 5′ end starts 524 nt upstream of the *amyA* stop codon | *Sbf*I |
| 1172-CHR-LR | CTAAATGTGCCCCCTCTACATTACTATAAC | 5′ end starts 24 nt upstream of *amyA* stop codon | n/a |
| 1172-CHR-RF | GTTATAGTAATGTAGAGGGGGCACATTTAG | complementary to 1172-CHR-LR | n/a |
| 1172-CHR-XmaI-RR | GCTAGCCCCGGGGCTTTAAATGGTGGAATATTGG | 3′ end starts 496 nt downstream of the *amyA* stop codon | *Xma*I |
